# German Children’s Use of Word Order and Case Marking to Interpret Simple and Complex Sentences: Testing Differences Between Constructions and Lexical Items

**DOI:** 10.1080/15475441.2015.1052448

**Published:** 2015-11-10

**Authors:** Silke Brandt, Elena Lieven, Michael Tomasello

**Affiliations:** ^a^ Department of Linguistics and English Language, Lancaster University; ^b^ School of Psychological Sciences, University of Manchester; ^c^ Department of Developmental and Comparative Psychology, Max Planck Institute for Evolutionary Anthropology

## Abstract

Children and adults follow cues such as case marking and word order in their assignment of semantic roles in simple transitives (e.g., *the dog chased the cat*). It has been suggested that the same cues are used for the interpretation of complex sentences, such as transitive relative clauses (RCs) (e.g., *that’s the dog that chased the cat*) (Bates, Devescovi, & D’Amico, 1999). We used a pointing paradigm to test German-speaking 3-, 4-, and 6-year-old children’s sensitivity to case marking and word order in their interpretation of simple transitives and transitive RCs. In Experiment 1, case marking was ambiguous. The only cue available was word order. In Experiment 2, case was marked on lexical NPs or demonstrative pronouns. In Experiment 3, case was marked on lexical NPs or personal pronouns.

Whereas the younger children mainly followed word order, the older children were more likely to base their interpretations on the more reliable case-marking cue. In most cases, children from both age groups were more likely to use these cues in their interpretation of simple transitives than in their interpretation of transitive RCs. Finally, children paid more attention to nominative case when it was marked on first-person personal pronouns than when it was marked on third-person lexical NPs or demonstrative pronouns, such as *der Löwe* ‘the-NOM lion’ or *der* ‘he-NOM.’ They were able to successfully integrate this case-marking cue in their sentence processing even when it appeared late in the sentence. We discuss four potential reasons for these differences across development, constructions, and lexical items. (1) Older children are relatively more sensitive to cue reliability. (2) Word order is more reliable in simple transitives than in transitive RCs. (3) The processing of case marking might initially be item-specific. (4) The processing of case marking might depend on its saliency and position in the sentence.

## Introduction

A large number of studies have investigated how children use language-specific cues in their sentence processing (Bates & MacWhinney, 1989). The vast majority of these studies have looked at children’s interpretation of simple transitives only. In the current study, we directly compare German children’s use of word order and case marking in their interpretation of simple transitives and transitive relative clauses (RCs). As has been suggested for Italian- and English-speaking adults (Bates, Devescovi, & D’Amico, 1999) and Japanese-speaking children (Suzuki, 2011), the same cues might be used for the interpretation of simple and complex sentences. However, our analysis of German child-directed speech indicates that cues show different degrees of reliability across constructions. In addition, especially in transitive RCs, nominative case is much more frequently marked on pronouns than on lexical NPs. Before presenting this corpus analysis and three experiments testing children’s use of cues in their interpretation of simple transitives and transitive RCs, we will discuss various factors that have been suggested to influence children’s sensitivity to cues within and across specific constructions.

### Cue availability and reliability across development

Adults’ sentence processing strategies are influenced by the availability and reliability of the pragmatic, semantic, prosodic, lexical, and morpho-syntactic cues provided in their language (e.g., Bates & MacWhinney, 1987; MacWhinney & Bates, 1989; Seidenberg & MacDonald, 1999; Trueswell & Tanenhaus, 1994). This is most evident in cross-linguistic comparisons. In English, for example, semantic role assignment is frequently and reliably signaled by word order and English-speaking adults basically always make use of this cue and tend to interpret the first NP in a sentence as referring to the agent. In languages such as Italian and German, on the other hand, word order is less reliable and adults make relatively more use of animacy, case marking, and/ or agreement to assign semantic roles (e.g., Kempe & MacWhinney, 1999; MacWhinney, Bates, & Kliegl, 1984). Although young children seem to use the same sentence-processing strategies as their caregivers from early on (e.g., Bates et al., 1984), they also show some divergent patterns (e.g., Chan, Lieven, & Tomasello, 2009; Dittmar, Abbot-Smith, Lieven, & Tomasello, 2008; Trueswell, Sekerina, Hill, & Logrip, 1999).

It has been suggested that adults are sensitive both to a given cue’s availability (how often this cue is present in the language and/ or in a specific construction) and its reliability (how often this cue points to the correct role assignment when present in the language and/ or in a specific construction), whereas young children are relatively more sensitive to a cue’s availability than to its reliability (e.g., MacWhinney, 2007; McDonald, 1986). In German child-directed speech, for example, both word order and case marking are available in more than 85% of all transitive sentences (Chan et al., 2009; Dittmar et al., 2008). That is, the vast majority of transitive sentences that German-speaking children hear contain (at least) two NPs and/ or (at least) one NP that is unambiguously marked for case. The word-order cue points to the correct role assignment in 79% of the sentences with two or more NPs, which means that in 79% of all transitive sentences that contain two or more NPs, the first NP refers to the agent. The case-marking cue points to the correct role assignment in 100% of the sentences that contain at least one NP with unambiguous case marking and is thus more reliable than the word-order cue. In accordance with these statistics, older German-speaking children and adults rely more on case marking than on word order when interpreting transitive sentences (Dittmar et al., 2008; Grünloh, Lieven, & Tomasello, 2011; Kempe & MacWhinney, 1999). Younger German-speaking children, however, are more likely to follow the word order cue than case marking. In Dittmar et al.’s (2008) study, when interpreting conflict sentences, where word order and case marking point to different semantic role assignments (e.g., *den Löwen miekt der Hund* ‘the-ACC lion is meeking the-NOM dog), children of mean age 2;6 and 5;0 were most likely to follow the word-order cue and assign the first NP to the agent role despite its accusative marking. Only at the age of 7;0 did children rely more on case marking than on word order and correctly assigned the second, nominative, NP to the agent role.

### Differences across lexical items

One possible explanation for why the younger children have not followed the more reliable case-marking cue in previous studies is that their processing of cue availability and reliability is initially only applied to those lexical items that are most frequently marked by or associated with a given cue. Many studies, for example, have suggested that, early in development, children tend to apply word-order rules to familiar, highly frequent lexical items only (e.g., Pine, Lieven, & Rowland, 1998; Tomasello, 2000). Similarly, Dittmar et al. (2008) showed that German-speaking children have much more experience with (nominative) case marking on pronouns (e.g., *er* ‘he-NOM’) than on definite NPs (e.g., *der Löwe* ‘the-NOM lion’). They suggested that the children in their study did not rely on case marking before the age of 7;0 because it was only marked on definite NPs. Whether German-speaking children can make use of case marking on pronouns before they understand case marking on definite NPs to assign agent-patient relations in transitive sentences has not been directly tested yet and will be investigated in the current study.

A study with Hebrew-speaking children suggests that they can make better use of case when it is marked on first-person personal pronouns rather than on lexical NPs, which are third person by default. Arnon (2010) tested 4-year-old Hebrew-speaking children’s comprehension of transitive RCs with lexical NPs or pronouns in the subject or object slot. In Hebrew, both nominative pronouns and lexical NPs are unmarked, whereas accusative pronouns and lexical NPs are marked. In the two conditions which are most relevant for the present paper, children had to assign agent-patient roles in object RCs with third-person lexical NPs in the subject slot (e.g., *what color are the shoes of the nurse that **the girl** is drawing*) and in object RCs with first-person pronouns in the subject slot (e.g., *what color are the shoes of the girl that **I**’m drawing*). Children were significantly better at comprehending the object RCs with first-person pronouns in the subject slot. Similar results were found in studies on German- and English-speaking children’s processing of RCs (Brandt, Kidd, Lieven, & Tomasello, 2009; Kidd, Brandt, Lieven, & Tomasello, 2007). Like Hebrew, German marks case on both pronouns and lexical NPs. In the current study, we will directly compare German-speaking children’s semantic role assignments in simple transitives and in transitive RCs with case being marked on lexical NPs, third-person demonstrative pronouns, or first-person personal pronouns. This will allow us to compare not only children’s use of case marking on first-person personal pronouns and third-person lexical NPs but also (1) their use of case marking on third-person lexical NPs and on third-person demonstrative pronouns, and (2) their use of case marking on third-person demonstrative pronouns and on first-person personal pronouns.

### Processing: Saliency and timing

Children’s sensitivity to pragmatic, semantic, prosodic, lexical, and morpho-syntactic cues also depends on their ability to detect the relevant cues and to integrate them in their online processing. The detection of morpho-syntactic cues depends on their saliency (Bates & MacWhinney, 1989). Some cues are hard to perceive and might thus not be picked up by young children. For example, MacWhinney, Pleh, and Bates (1985) have shown that in Hungarian the accusative marker −*t* after consonant clusters is learned later than the same marker appearing after strong vowels. In German, case is mainly marked on determiners and pronouns.^1^ Determiners are usually unstressed. So, children might not pay much attention to the different case endings in sentences such as *de**r** Löwe jagt de**n** Hund* ‘the-**NOM** lion is chasing the-**ACC** dog’ (cf. Szagun, 2004). Picking up case endings on unstressed determiners is not an easy task and it takes German-learning children some years to comprehend and produce the correct forms in all contexts (e.g., Clahsen, 1984; Dittmar et al., 2008; Szagun, 2004). Case marking on personal pronouns, on the other hand, tends to be more salient. For example, the nominative form *er* ‘he’ is easy to distinguish from the accusative form *ihn* ‘him’.

Finally, after children have cracked the case marking system and started to process the availability and reliability of the different cues in their language, they also need to be able to use these cues online. In most studies, children are asked to interpret so-called conflict sentences, where cues point to different interpretations, such as *him threw the ball or *the dog chase the horses. These studies have found that young children tend to be better at using local cues than distributed cues (cf. Slobin, 1982). Local cues, such as case marking, can be used on the spot. For example, once a German-speaking child has acquired the case marking system and encounters a noun with unambiguous case marking, she can assign a semantic role to it without processing the other nouns or verbs in the sentence. Distributed, or global, cues, such as word order or agreement, on the other hand, can only be used after the whole sentence has been processed. Agreement, for example, is only informative after all NPs and VPs have been processed. It should be noted, however, that children tend to acquire and use word order, which is a global cue, before they reliably use case marking—a local cue (e.g., Dittmar et al., 2008).

Moreover, it has been shown that, even in the absence of clear case marking or other local cues, children and adults do make semantic and syntactic role assignments before the whole sentence has been processed. This is most evident in garden-path phenomena, which are found in both children’s and adults’ sentence processing. They show that children and adults use pragmatic, semantic, prosodic, lexical, and morpho-syntactic cues as they process sentences in a linear fashion (i.e., word-by-word) even though most of these cues are not fully reliable (e.g., Altmann & Kamide, 1999; MacDonald, 1999; Tanenhaus & Trueswell, 1995; Trueswell et al., 1999). The difference between children and adults seems to be that adults can revise their initial semantic and syntactic role assignments if they turn out to be wrong as more or all arguments and verbs of the sentence are being processed. Children, on the other hand, have trouble revising their initial semantic and syntactic role assignments, especially when the crucial cue, which signals the correct interpretation, appears late in the sentence (Choi & Trueswell, 2010; Kidd, Stewart, & Serratrice, 2011; Trueswell et al., 1999). For example, a study by Choi and Trueswell (2010) suggests that children will only make use of lexical cues when they are presented early in the sentence. According to Choi and Trueswell, children’s difficulty in reinterpreting a sentence based on cues appearing late in the sentence is due to their limited cognitive control.

In summary, previous research on children’s acquisition and processing of cues in simple sentences suggests that (1) young children are relatively more sensitive to cue availability than to cue reliability; (2) their processing of cues might initially be item-specific; (3) their processing of cues depends on the cues’ saliency; (4) especially young children are more likely to use local cues, such as case marking, rather than global cues, such as agreement; and (5) their sentence processing is probabilistic and incremental, they have difficulties recovering from misinterpretations, and they have difficulties integrating cues that come in late in the sentence.

### Processing of cues across constructions

What does this all mean for children’s processing of complex sentences? Few studies have been done to directly compare the acquisition and processing of cues across constructions. Looking at 5- to 7-year-old Japanese-speaking children, Suzuki (2011) found that children who reliably make use of case marking in their interpretation of simple transitives also use case marking to interpret transtive RCs. Japanese has SOV word order and prenominal RCs. This means that subject RCs display a noncanonical OVS word order. As is the case for German object RCs, the correct patient-first interpretation is signaled by case marking (see example 1). Suzuki (2011) found that the children who reliably used case marking to correctly interpret simple OV patterns (see example 2) were also able to use case marking to correctly interpret the non-canonical OVS pattern in subject RCs.

Subject RC

1. [*kuma-o* *hikkaita*] *panda*
  bear-ACC scratched panda
  ‘The panda which scratched a bear’

Simple transitive

1. *Kuma-o* *hikkakimasita*.
  bear-ACC scratched
  ‘(The panda) scratched the bear’

Similarly, for adults, Bates et al. (1999) have argued that the processing of cues is basically the same for simple and complex sentences. They showed that English-speaking adults overwhelmingly follow word order and Italian-speaking adults mainly follow agreement in their interpretation of both simple transitives and embedded RCs. Interestingly, however, processing times suggest that Italian adults struggled more with conflicting information in complex sentences than in simple sentences. Their processing of main clauses and RCs in complex sentence structures was significantly slowed down when word order and agreement were in conflict, that is, when the second noun agreed with the verb. But this was not the case for their processing of simple sentences.

When interpreting simple sentences with conflicting cues, Italian-speaking children start to reliably follow agreement over word order and animacy at the age of 7;0 (e.g., when hearing a sentence such as ‘the dog chase the motorbikes,’ they choose ‘motorbikes’ as the agent) (Devescovi et al., 1999). But it takes them another two years until they start to correctly interpret RCs with conflicting word-order, animacy, and agreement information, such as ‘the baker watches the mouse that are chasing the cats,’ where the final NP ‘the cats’ needs to be interpreted as the agent (Arosio, Guasti, & Stucchi, 2011). Note that these sentences are fully grammatical in Italian. In addition, Arosio and colleagues (Arosio et al., 2011; Arosio, Yatsushiro, Forgiarini, & Guasti, 2012) found that the successful processing of (conflicting) cues in RCs depends on children’s short-term memory capacity and possibly on the position of the cue in the sentences. For example, in their 2012 study of German, Arosio et al. found that children with relatively good short-term memory (measured by digit-span) were better at integrating agreement information in conflict with word order than children with medium and low digit spans. That is, only high digit-span children at the age of 7;0 correctly interpreted sentences like (3).

1. *die* *Fee*, *die* *die* *Polizisten* *geschoben* ***haben***.
  the fairy who the police men pushed  **have-PL**
  ‘the fairy who the police men have pushed’

This sentence is completely ambiguous before the auxiliary is encountered in sentence-final position. According to word order, the first NP (‘the fairy’) is the subject/ agent. The auxiliary, however, agrees with the second NP (‘the policemen’). With singular masculine nouns, which are unambiguously marked for case, the disambiguating information is provided earlier in the sentence.

1. *die* *Fee*, *die* ***der***   *Polizist*  *geschoben* *hat*.
  the fairy who **the-NOM** police man pushed  has-SG
  ‘the fairy who the-NOM police man has pushed’

In this case, 7-year-old children with medium digit spans were also able to correctly interpret the sentence. Children with low digit spans still had problems integrating case marking when it conflicted with word order. Overall, children correctly interpreted only 58.6% of the object RCs that were disambiguated by case, such as (4), and 48.9% of the object RCs that were disambiguated by agreement, such as (3). These results suggest that cues that appear late in the sentence require more memory resources and are harder to integrate than cues that come earlier in the sentence (for similar results on adults’ processing of case and agreement in German RCs, see Friederici, Steinhauer, Mecklinger, & Meyer, 1998). The positioning and timing of the cues might also explain why Suzuki (2011) did not find a difference between Japanese children’s comprehension of simple OV(S) structures and their comprehension of complex OVS structures. In Japanese, in both simple and complex OV(S) structures, the patient-first interpretation is signaled by the case marking on the first NP. In Arosio et al.’s (2012) experiment on transitive RCs, on the other hand, only the second NP displayed the crucial case marking (see example (4)). In Dittmar et al.’s (2008) study on simple transitives, the first NP was marked for accusative case (e.g., *den Löwen miekt der Hund* ‘the-ACC lion is meeking the-NOM dog’) and they found that German-speaking 7-year-olds correctly interpreted 69% of the simple OVS structures compared with 58.6% reported for object RCs (Arosio et al., 2012).

Note, however, that case marking might also be easier to process than agreement because it is a local cue, which, unlike agreement, can be used on the spot (Slobin, 1982). This assumption is supported by a study by Guasti, Stavrakaki, and Arosio (2012), which tested Greek children’s sensitivity to agreement and case marking in their interpretation of RCs. For example, in an object RC like ‘the horse that are chasing the lions’, agreement indicates that the embedded NP ‘lions’ needs to be interpreted as the agent. In an object RC like ‘the monkey that is washing the-NOM bear,’ case marking indicates that the embedded NP ‘bear’ needs to be interpreted as the agent. The agreement cue is encountered earlier in the sentence than the case-marking cue. Nevertheless, in their interpretation of object RCs children were better at interpreting sentences that were disambiguated by case than sentences that were disambiguated by agreement (Guasti et al., 2012).

To summarize, the studies on children’s (and adults’) processing of cues in complex sentences suggest that there is a prolonged problem with conflict sentences. This might be caused by the fact that complex sentences often display different word orders, so that the position of the cues is not the same as in simple transitives. Some studies also suggest that especially cues that appear late in the sentence are hard to process and to integrate (e.g., Choi & Trueswell, 2010).

## Corpus study: Cues in transitive relative clauses in German child-directed speech

In order to see whether the transitive RCs that German-speaking children hear in their input display the same availability and reliability rates for case marking and word order that have been reported for transitive sentences in general (see Dittmar et al., 2008), we looked at child-directed speech in three different German corpora that are available on CHILDES (MacWhinney, 2000). For some of the transcripts from the Leo corpus (Behrens, 2006), child-directed speech has been tagged.^2^ This allowed us to use CLAN and search for utterances containing two verbs. These two-verb utterances were then searched by hand. On average, we found one transitive RC per transcript and got a list of 85 transitive RCs produced by Leo’s caregivers, when the child was between the age of 2;0 and 5;0. Speech addressed to six children from the Szagun (2001) corpus had already been coded for construction types by Stoll, Abbot-Smith, and Lieven (2009). We had two coded transcripts per child, which allowed us to extract 11 transitive RCs produced by the mothers, when their children were 1;8 and 2;5. Finally, the transcripts from three children between the age of 2;6 and 8;0 from the Rigol corpus have been searched by hand. This search gave us a list of 226 transitive RCs produced by the mothers. In the end, we were able to analyze 322 transitive RCs produced by 10 different German caregivers.

First, we determined the word order of these transitive RCs. Note that, unlike in English, NNV patterns in German can be interpreted as either SOV or OSV, if case marking, agreement-, or semantic-pragmatic cues are absent:

1. *Da*  *ist* *das* *Pferd*, *das* *die* *Kuh* *schubst*.
  There is the horse that the cow pushes
  ‘there’s the horse that is pushing the cow/ that the cow is pushing’

However, based on case marking, agreement, and/ or semantic-pragmatic cues, all transitive RCs in our sample of child-directed speech could be categorized as either SOV or OSV. In example (6) from the Leo corpus, for instance, the decision could be made on the basis of semantic information:

1. Auch ein Wort, was Leo furchtbar gerne anwendet. 
  also a word that Leo terribly  gladly uses
  ‘also a word that Leo really likes to use’

321 of the 322 transitive RCs contain both a relative pronoun and an embedded NP (e.g., *was* ‘that’ and *Leo* in example (6)). Word order is thus available in almost 100% of all transitive RCs. However, the majority of transitive RCs display an object-first order (OSV). Only 22% (70/321) of the transitive RCs that contain a relative pronoun and an embedded NP are SOV. Based on the assumption that the default word order of German is SO, we can conclude that word order has a reliability and validity of only 0.22 in transitive RCs.^3^ Recall that Dittmar et al. (2008) looked at both simple and complex transitive constructions in German child-directed speech and found that, across all transitive constructions, word order has a validity of 0.68. In general, most transitive sentences that German-speaking children hear are SO. However, based on these corpus data, we could also suggest that syntactic cues should be calculated on a construction-specific level and our experiments will investigate whether and at what age German-speaking children process syntactic cues on a construction-specific or on a more global level.

Within transitive RCs, case marking can be provided on the relative pronoun, as in (7), and/or on the embedded NP, as in (8).

1. Kaiser Wilhelm, **den**   die Oma  auch hat.
  Emperor Wilhelm **that-ACC** the grandma also has 
  ‘Emperor Wilhelm (train) that grandma has, too’
2. seine Trambahn, die **er**   muehsam ausgeschnitten hat. 
  his  tram   that **he-NOM** painfully cut out   has
  ‘his tram that he has painfully cut out’

Overall, 77% (247/322) of all transitive RCs in our sample of child-directed speech show case marking on the relative pronoun and/ or the embedded NP. Since case marking is 100% reliable, the validity for case marking in transitive RCs is similar to the validity of case marking in transitive clauses in general (i.e., 86%; Dittmar et al., 2008). However, only 15% (49/322) of all relative pronouns are unambiguously marked for case. On the other hand, 69% (222/322) of all embedded NPs are marked for case and most of these (207/222) are marked nominative, as in (8). Taking a closer look at these nominative embedded NPs, it turns out that the vast majority (182/207) are nominative personal pronouns.

To summarize, the word-order cue is far less reliable and valid in transitive RCs than has generally been observed for transitive clauses. In other words, most transitive RCs are actually OSV. The case-marking cue shows validity that is similar to what has been reported for simple and complex transitives (Dittmar et al., 2008). However, in transitive RCs, case marking is provided rather late in the sentence (i.e., on the embedded NP) and it most often comes in the form of nominative personal pronouns, such as *du* ‘you-NOM’. Based on these and earlier corpus findings and experimental studies, we can now make the following predictions.

### Availability and reliability of word order and case across age and constructions

It has been suggested that older children and adults are sensitive both to a given cue’s availability and its reliability, whereas young children are relatively more sensitive to a cue’s availability than to its reliability (e.g., Dittmar et al., 2008; MacWhinney, 2007; McDonald, 1986). Our corpus study has shown that the availability of case marking and word order is similar across simple transitives and transitive RCs. The reliability of case marking is also similar across constructions, but word order is much more reliable in simple transitives than in transitive RCs. Based on these assumptions and observations, we should predict that the older children would be more likely to follow case marking, especially in their interpretation of transitive RCs. The younger children might follow both case marking and word order in their interpretation of both constructions, which will lead to at-chance performance when they have to interpret conflict sentences in which the two cues are competing with each other.

### Lexical specificity and saliency of case marking

However, our corpus study has also shown that case marking in transitive RCs is mostly provided on a handful of personal pronouns and Dittmar et al. (2008) have reported similar patterns for simple transitives. Furthermore, case marking on personal pronouns is more salient and easier to detect than case marking on lexical NPs or demonstrative pronouns. Forms like *er* ‘he-NOM’ and *ihn* ‘him-ACC’ are easier to distinguish than *der Hund* ‘the-NOM dog’ and *den Hund* ‘the-ACC dog’ or *der* ‘he-NOM’ and *den* ‘he-ACC.’ This suggests that even the older children might be more likely to follow case when it is marked on personal pronouns than when it is marked on lexical NPs or demonstrative pronouns.

### Position and timing of case marking

Finally, even though case marking is much more reliable than word order in transitive RCs, children might not always be able to integrate this cue in their online processing of complex sentences. Even the older children might find it easier to integrate case marking in their processing of simple transitives than in their processing of transitive RCs because the cue is provided earlier in the sentence (Choi & Trueswell, 2010). Moreover, the use of case marking in the interpretation of transitive RCs can be very difficult because the case marking on the relative pronoun following the head NP can conflict with the case marking on the determiner preceding the head NP. This issue will be investigated in Experiment 3.

### Experiment 1: Simple transitive and transitive RCs with no case marking on lexical NPs or demonstrative pronouns

The first experiment was set up to investigate how German-speaking children use word order in their interpretation of simple transitives and transitive RCs. For this purpose, children were asked to interpret sentences without clear case marking. The sentences contained only neuter and feminine nouns, which have the same form in the nominative and accusative (see example sentences in Table 2).

#### Participants

We tested two age groups; 24 monolingual German 3-year-olds (mean = 3;0, range: 2;11-3;2) and 16 monolingual 6-year-olds (mean = 6;8, range: 6;6-6;11) were included in the study. Another 11 children were tested but were excluded from the main analysis due to experimenter error (4), side bias (3, always pointed to the same side), fussiness (3), or problems in naming the animals occurring in the test sentences before the test (1). The 3-year-olds were recruited from nurseries in a midsize German city and tested in a quiet room in their nurseries. The 6-year-olds were recruited from a database of families who volunteered to take part in psycholinguistic studies and were tested in a quiet room at a research institute. None of the children had any known language impairments.

#### Materials

Twenty-four test sentences were constructed. The first manipulation was construction type (simple transitive vs. transitive RC). The second manipulation was form of the second NP in the simple transitives or the embedded NP in the RCs (lexical NP versus demonstrative pronoun). These manipulations resulted in four conditions (see Table 2). All noun phrases were expressed by feminine or neuter nouns, which have the same form in the nominative and the accusative. Thus, case marking was not available. All test sentences could, in principle, be interpreted as either subject-first (simple transitive: SVO; RC: SOV) or object-first (simple transitive: OVS; RC: OSV). In Experiments 1 and 2, we used third-person demonstrative pronouns (*die* ‘she/that’, *das* ‘it/this’) rather than third-person personal pronouns (*sie* ‘she’, *es* ‘it’) because third-person demonstrative pronouns are more frequent than third-person personal pronouns in German child-directed speech and German-speaking children’s own production. A corpus analysis also suggests that these demonstrative pronouns are more likely to refer to subjects than to objects (see Table 1).

**Table 1.** Use of demonstrative pronouns in German (Leo corpus).

	Input	Child
***die*-SG SUBJECT**	815	2329
***die*-SG OBJECT**	413	691
***das*-SG SUBJECT**	5803	6268
***das*-SG OBJECT**	2275	2051

**Table 2.** Conditions and example sentences for simple transitives and transitive RCs with no case marking on lexical NPs or demonstrative pronouns.

trans NN	Das Pferd schubst jetzt die Kuh. ‘the-NOM/ACC horse pushes now the-NOM/ACC cow’
rel NN	Das Pferd, das die Kuh schubst. ‘the-NOM/ACC horse who-NOM/ACC the-NOMACC cow pushes’
trans NPro	Das Pferd schubst die jetzt mal. ‘the-NOM/ACC horse pushes she/her now’
rel NPro	Das Pferd, das die jetzt schubst. ‘the-NOM/ACC horse who-NOM/ACC she/her now pushes’

We had six test sentences per condition. ‘Trans’ stands for simple transitive and ‘rel’ stands for relative clause. ‘NN’ stands for sentences with two lexical NPs and ‘NPro’ stands for sentences where the second NP or the embedded NP is expressed by a third-person demonstrative pronoun.

In order to make the RCs propositionally and syntactically as simple as possible and comparable to the simple transitives, they were all right-branching, that is, not center-embedded and attached to isolated head NPs. This type of RC is commonly produced by young German-speaking children (Brandt, Diessel, & Tomasello, 2008). All test sentences were matched for length by adding *jetzt* ‘now’ and *mal* ‘PARTICLE’ in some conditions, so that all items contained six words.

The test sentences were presented together with 24 movie pairs. In the test phase, the children always saw the two movies of a pair simultaneously. The two movies of a pair only differed in semantic role assignment. For example, the horse would push the cow in one movie while the cow would push the horse in the other. We used 12 familiar animals and six familiar transitive verbs/actions for the movies. The animals were camel, cat, cow, crocodile, kangaroo, giraffe, horse, mouse, pig, seal, turtle, and zebra. The actions were feed, push, comb, tickle, stroke, and wash. Two animals never occurred together more than once, and each animal was the actor of each action once.

Eight experimental orders were created by randomly matching conditions with movie pairs. Three of the 3-year-olds and two of the 6-year-olds were tested with each list. The movies played on the left and right side of a laptop computer screen (23cm x 37cm). The default subject-first (SVO/SOV) interpretation of the ambiguous sentences appeared on both sides equally often.

### Procedure

In Experiments 1 and 2, we mainly followed the procedure used by Dittmar et al. (2008). The animal actors (hand puppets) from the movies were brought to the experimental sessions. At the beginning of the session, the experimenter asked the child to name each animal. Most children could label all animals. One 3-year-old had to be excluded because he could only name a few. In the following warm-up task the children practiced the pointing task. The experimenter presented them with picture pairs of familiar objects (cow-duck, ball-house, car-tree, fish-pig), on the laptop screen and asked them to point to the correct one by saying, for example, *zeig mir mal das Bild: das ist die Ente* ‘show me the picture: that’s the duck.’

After the pointing practice, the children were familiarized with the movies. A sample of six movie pairs was selected, so that the children saw each of the six actions and each of the twelve animals before the test trials. In the familiarization trials, the movies were not played simultaneously, but individually, after one another. The side (left or right) where the children saw the first movie of a pair was counterbalanced within subjects. Each movie was played for 10 seconds and the experimenter described the action: *guck mal, das ist schubsen* ‘look that’s pushing.’ Then the children were asked to label the animals in the still picture. Most children could name all animals on the screen. When a child did not name one of the animals, the experimenter told the child the name again and asked the child to repeat it.

Then the test trials were presented in one block. Before the movies that were described by test sentences containing demonstrative pronouns, the experimenter showed the child the animal actor or patient that the pronoun referred to and asked for the name, for example, ‘the cow,’ again. Then she said, for example: *lass uns mal gucken, was hier gleich mit der Kuh kommt* ‘let’s see what’s happening with the-DAT cow now.’ Importantly, the animal was introduced in dative case and never in the nominative or accusative. Before each test trial, a red center point drew the child’s attention to the center of the laptop computer screen. Then the child saw the two scenes (e.g., horse pushing cow and cow pushing horse). The child saw two movies from a pair simultaneously and heard the test sentence, which was prerecorded (e.g., *guck mal, das Pferd schubst jetzt die Kuh* ‘look, the-NOM/ACC horse pushes now the-NOM/ACC cow’). The movies were played for 10 seconds, and the children heard the test sentence twice. After the movies had stopped, the experimenter asked the child to point to the correct still picture by saying *zeig mir das Bild: das Pferd schubst jetzt die Kuh* ‘show me the picture: the-NOM/ACC horse pushes now the-NOM/ACC cow.’ The still pictures were chosen so that the action was clearly visible.

If the child did not point, the experimenter repeated the live prompt a second time. The children’s pointing behavior was coded live by the experimenter and recorded by a camera that stood behind the children. All live codes were double-checked with the video recordings. The children pointed either to one of the still pictures or to both. Points to both pictures were coded as ambiguous.

### Results experiment 1

**Figure 1.**
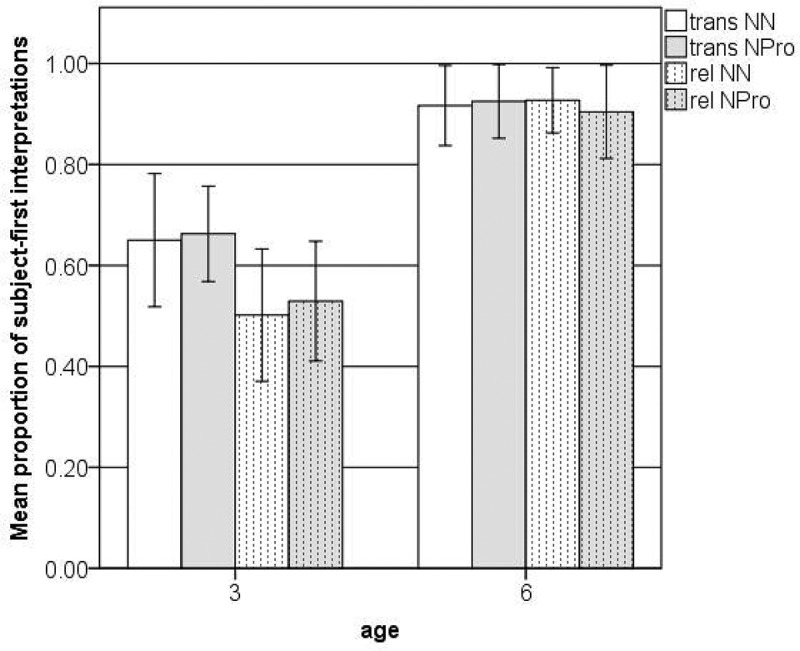
Subject-first interpretations of simple transitives (trans) and transitive RCs (rel) with no case marking on lexical NPs (NN) or demonstrative pronouns (NPro) (Experiment 1).

The ambiguous sentences we used in Experiment 1 can be interpreted as either subject-first (simple transitive: SVO; RC: SOV) or object-first (simple transitive: OVS; RC: OSV). German-speaking adults have a strong tendency to read these sentences as subject-first (see MacWhinney et al., 1984; Nitschke, Kidd, & Serratrice, 2010). Figure 1 shows the percentage of ambiguous sentences that the children interpreted in this adult-like way (i.e., as subject-first) in each condition. For this calculation we only considered trials with unambiguous responses. Out of 960 trials 139 were excluded because children only gave ambiguous or no responses. Many of these ambiguous responses came from two particular three-year-olds, who almost never gave a clear response.

To investigate the patterns in Figure 1, the data were analyzed using Generalized Linear Mixed Models (GLMM) (Baayen, Davidson, & Bates, 2008; Jaeger, 2008) with the lme4 package for Linear Mixed Effects (Bates & Maechler, 2010) in R. For all analyses presented below, we used backward stepwise comparison by removing main factors and two-way interactions to establish which factors and interactions contribute to the goodness of fit of the model. First we analyzed the data from both age groups together, testing for the fixed effects of age (3 vs. 6), construction type (simple transitive vs. transitive RC), and form of NP (lexical NP vs. demonstrative pronoun) and the random effects of subjects and items against a null model. The final model showed main effects for age and construction type (see Table 3).^4^

Next we fitted two separate models for the 6- and 3-year-olds, testing for all main effects and two-way interactions. For the 6-year-olds, the backwards selection eliminated all main factors and two-way interactions resulting in a null model. As can be seen in Figure 1, the 6-year-olds had a clear preference to interpret all ambiguous sentences as subject-first. In the present study, they used the word-order cue and had no support from case marking or other cues, such as animacy or agreement, and they applied it to both simple transitives and transitive RCs. In addition, whether the second or embedded NP was expressed by a lexical NP or by a demonstrative pronoun did not influence the older children’s interpretations.

The final model for the three-year-olds showed a main effect for construction type only (see Table 4). As can be seen in Figure 1, the younger children were more likely to interpret simple transitives without case marking or agreement as subject-first (SVO) than to interpret ambiguous RCs as subject-first (SOV). They interpreted most simple transitives as subject-first. However, unlike the older children, who also reliably used the word-order cue in their interpretation of transitive RCs, the younger children showed no clear preference in their interpretation of the complex sentences. The younger children were above chance in their subject-first interpretation of the trans NPro sentences (Wilcoxon *p* = .002) and at chance in all other conditions. This might suggest some item specificity in the younger children’s interpretation of simple transitives. That is, they were more likely to interpret simple transitives as SO when the second NP was expressed by a demonstrative pronoun than when it was expressed by a lexical NP. However, as for the older children, the form of the second or embedded NP did not turn out significant in the GLMM.

**Table 3.** GLMM main effects Experiment 1. Subject-first interpretations of simple transitives and transitive RCs with no case marking on lexical NPs or demonstrative pronouns.^5^

Parameter	Estimate	Std. Error	z-statistic	*p*-value
Intercept	0.124	0.222	0.557	0.577
AGE (6 vs. 3)	2.470	0.341	7.238	<0.0001
CONSTRUCTION TYPE (trans vs. rel)	0.502	0.181	2.776	0.006

**Table 4.** GLMM main effects 3-year-olds Experiment 1. Subject-first interpretations of simple transitives and transitive RCs with no case marking on lexical NPs or demonstrative pronouns.^6^

Parameter	Estimate	Std. Error	z-statistic	*p*-value
Intercept	−0.015	0.186	−0.081	0.935
CONSTRUCTION TYPE (trans vs. rel)	0.606	0.199	3.048	0.0023

If we leave in the trials with ambiguous or null responses, we find the exact same patterns of results for both age groups. The random subject effect variance is at 2.227 for the older children and at 0.325 for the younger children. The relatively high subject variance for the older children is probably due to the fact that three of the 16 six-year-olds only interpreted around 67% of the test items as subject-first, whereas the rest of the older children interpreted at least 92% of the test items as subject-first.

### Discussion experiment 1

The 6-year-olds clearly followed word order in their interpretation of both simple transitives and transitive RCs. In the absence of any additional cues, such as animacy, case, or agreement, both constructions are interpreted as subject-first. The same results have been found for adults (e.g., MacWhinney et al., 1984; Nitschke et al., 2010) and it has been suggested that the word order cue in German is based on the configuration of the two nouns, rather than the configuration of the nouns and the verb (Kempe & MacWhinney, 1998). The 3-year-olds, however, were more likely to apply the word-order rule to simple transitives than to transitive RCs. This supports our assumption that, if children’s sensitivity to the reliability of cues is construction-specific, they will pay more attention to word order when processing simple transitive sentences than when processing transitive RCs. The corpus study has shown that, unlike simple transitives, the majority of transitive RCs that German-speaking children hear are actually object-first. Our results therefore suggest that younger children process and apply the word-order cue on a more local, construction-specific level (Wittek & Tomasello, 2005). In Experiment 2 we tested whether, early in development, the processing of case marking is also construction-specific and whether it is item-specific, that is, whether children find it easier to follow case marking on demonstrative pronouns than on lexical NPs.

## Experiment 2: Simple transitives and transitive relative clauses with case marking on lexical NPs and demonstrative pronouns

In Experiment 2, we tested another group of German-speaking children with simple transitive and transitive RCs with clearly case-marked NPs. The case marking signaled either a subject-first (SVO/ SOV) or an object-first (OVS/ OSV) reading. In order to correctly interpret the object-first sentences, children had to use the case-marking cue without support from the word-order cue or any other cues. Based on the results from other studies that tested German children’s comprehension of object-first sentences (e.g., Dittmar et al., 2008; Grünloh et al., 2011), we hypothesized that the older children, but not the 3-year-olds, could make use of the case-marking cue when it is not supported by any other cues. Furthermore, we wanted to investigate whether the older children use case marking to comprehend both simple transitives and transitive RCs. Based on cue reliabilities and validities, we should not expect any differences between these two constructions. However, as discussed above, in transitive RCs the case-marking cue appears relatively late in the sentence, and it has been suggested that children are not very good at processing and integrating cues that appear late in the sentence (Choi & Trueswell, 2010). Therefore, the older children might also be better at using case when processing simple transitives. In addition, it has been shown that the processing of (nominative) case is easier when it is provided on pronouns (e.g., Arnon, 2010). Therefore, we expect both three- and six-year-old children to be better at integrating case-marked pronouns in their sentence processing than case-marked lexical NPs. This should be the case for both simple transitives and transitive RCs.

### Participants

Twenty-four monolingual German three-year-olds (mean = 3;0, range: 2;11-3;2) and 24 monolingual 6-year-olds (mean = 6;6, range: 6;3-6;10) were included in Experiment 2. Another 12 children were tested but were excluded from the main analysis due to experimenter error (4), side bias (3 almost exclusively pointed to one side: 22/24, 23/24, and 24/24), fussiness (3), or problems in naming the animals before the test (2). The 3-year-olds were recruited from nurseries in a midsize German city and tested in a quiet room in their nurseries. The 6-year-olds were recruited from a database of families who volunteered to take part in psycholinguistic studies and were tested in a quiet room at a research institute. None of the children had any known language impairments.

### Materials

Twenty-four test sentences were constructed. The first manipulation was construction type (simple transitive vs. transitive RC). The second was word order (SO versus OS). The third was form of the second NP in simple transitives or the embedded NP in transitive RCs (lexical NP versus demonstrative pronoun). These manipulations resulted in eight conditions (see Table 5). As in Experiment 1, we used third-person demonstrative pronouns because, overall, they are more frequent than third-person personal pronouns. In the Leo corpus (Behrens, 2006), the masculine nominative demonstrative *der* ‘he-NOM’, for example, occurs more than 3,500 times in the child’s speech, whereas the masculine nominative personal pronoun *er* ‘he-NOM’ only occurs a bit more than 800 times.

**Table 5.** Conditions and example sentences for simple transitives and transitive relative clauses with case marking on lexical NPs and demonstrative pronouns.

trans SO	Der Hase schubst jetzt den Vogel. ‘the-NOM rabbit pushes now the-ACC bird’
rel SO	Der Hase, der den Vogel schubst. ‘the-NOM rabbit who-NOM the-ACC bird pushes’
trans SPro	Der Hase schubst den jetzt mal. ‘the-NOM rabbit pushes him now’
rel SPro	Der Hase, der den jetzt schubst. ‘the-NOM rabbit who-NOM him now pushes’
trans OS	Den Vogel schubst jetzt der Hase. ‘the-ACC bird pushes now the-NOM rabbit’
rel OS	Der Vogel, den der Hase schubst. ‘the-NOM bird who-ACC the-NOM rabbit pushes’
trans OPro	Den Vogel schubst der jetzt mal. ‘the-ACC bird pushes he now’
rel OPro	Der Vogel, den der jetzt schubst. ‘the-NOM bird who-ACC he now pushes’

We had three test sentences per condition, resulting in 24 test items. ‘Trans’ stands for simple transitive and ‘rel’ for transitive RC. ‘SO’ stands for subject-first sentences with two lexical NPs. ‘SPro’ stands for subject-first sentences where the second or embedded NP, that is, the object, is expressed by a demonstrative pronoun. ‘OS’ stands for object-first sentences with two lexical NPs, and OPro stands for object-first sentences where the second or embedded NP, that is, the subject, is expressed by a demonstrative pronoun.

As in Experiment 1, the RCs were all right-branching, that is, not center-embedded, and were attached to isolated head NPs. All test sentences were matched for length by adding *jetzt* ‘now’ and *mal* ‘PARTICLE’ in some conditions, so that all test items contained six words. To ensure clear case marking on all nouns, the animal names used in the test sentences in Experiment 2 have masculine gender in German. The animals were rabbit, monkey, tiger, bear, lion, dog, elephant, frog, donkey, tomcat, hedgehog, and bird. The actions were the same as in Experiment 1: feed, push, comb, tickle, stroke, and wash.

### Procedure

The procedure was the same as in Experiment 1. Eight experimental orders were created by randomly matching conditions with movie pairs. Three children of each age group were tested with each list. For each movie pair we counterbalanced which particular movie correctly matched the test sentence. For example, for the pair ‘rabbit feed monkey’ and ‘monkey feed rabbit,’ for half of the children the test sentence described ‘rabbit feed monkey,’ and for the other half the test sentence described ‘monkey feed rabbit.’ The movies played on the left and right side of a laptop computer screen (23cm x 37cm). Within each order, the target, that is, the movie described by the test sentence, appeared on both sides equally often (12 times on the right and 12 times on the left). The same side was never the correct choice more than twice in a row. In none of the experimental orders did the correct choice alternate regularly (e.g., LRLRLRLR). The first point after the test sentence (the live prompt) was coded as correct or incorrect. There were no ambiguous points (i.e., pointing to both pictures) in Experiment 2.

### Results experiment 2

**Figure 2.**
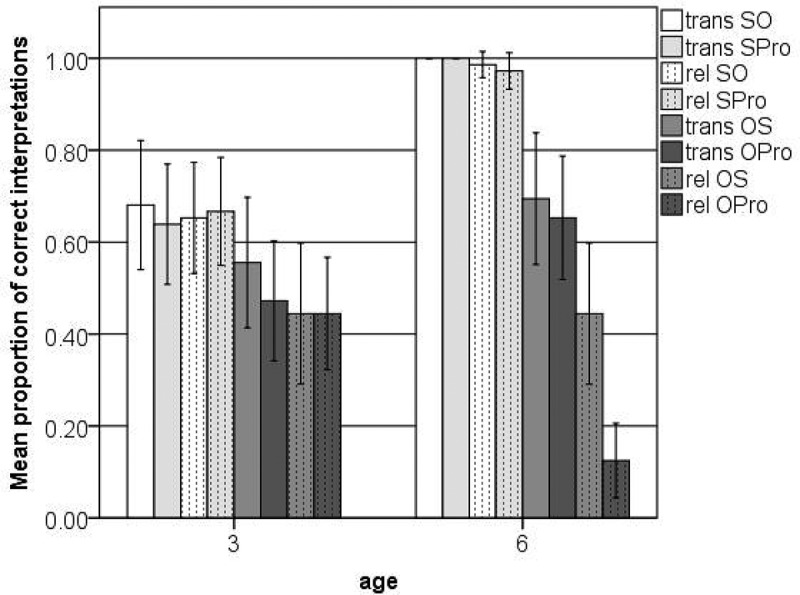
Correct interpretations of simple transitives (trans) and transitive relative clauses (rel) with case marking on lexical NPs (SO, OS) and demonstrative pronouns (SPro, OPro) (Experiment 2).

Figure 2 shows the percentage of correct interpretations of all trials in each condition. The GLMM analysis procedure was the same as for Experiment 1. First we analyzed the data from both age groups together, testing for the fixed effects of age (3 vs. 6), order (SO vs. OS), construction type (simple transitive vs. transitive RC), and form of NP (lexical NP vs. demonstrative pronoun) as well as the random effects of subjects and items against a null model. The random item effect showed zero variance and was removed from the model. The final model showed main effects for order and age as well as interactions between order and age, construction type and age and form of NP and age (see Table 6).

**Table 6.** GLMM main effects and 2-way interactions Experiment 2. Correct interpretation of simple transitives and transitive relative clauses with case marking on lexical NPs and demonstrative pronouns.

Parameter	Estimate	Std. Error	z-statistic	*p*-value
Intercept	−0.077	0.215	−0.357	0.721
ORDER (SO vs. OS)	0.795	0.175	4.54	<0.0001
AGE (6 vs. 3)	−0.583	0.334	−1.743	0.081
CONSTRUCTION TYPE (trans vs. rel)	0.156	0.174	0.894	0.371
NP (pro vs. N)	−0.156	0.174	−0.894	0.371
ORDER:AGE	4.652	0.668	6.959	<0.0001
AGE:CONSTRUCTION TYPE	1.792	0.327	5.475	<0.0001
AGE:NP	−0.803	0.323	−2.486	0.0129

Next we did separate analyses for the 3- and 6-year-olds, testing for all main effects and two-way interactions. The final model for the older children showed main effects for order, construction type, and form of NP, as well as a significant interaction between construction type and form of NP.

The interaction between order and construction type could not be interpreted because of extreme floor and ceiling effects (see Figure 2). However, when we only analyzed the object-first sentences for the 6-year-olds, we found main effects for construction type and form of NP as well as a significant interaction between construction type and NP.

As indicated by the main effect for order in Table 7, the 6-year-olds performed at ceiling in all conditions where the subject precedes the object (i.e., trans SO, rel SO, trans SPro, and rel SPro). In these sentences, word order and case marking point to the same interpretation. The form of the second NP in the simple transitives and the embedded NP in the transitive RCs did not affect the older children’s performance; that is, whether accusative case was provided on lexical NPs or demonstrative pronouns did not affect the 6-year-olds’ performance on these sentences.

**Table 7.** GLMM main effects and 2-way interactions 6-year-olds Experiment 2. Correct interpretation of simple transitives and transitive relative clauses with case marking on lexical NPs and demonstrative pronouns.^7^

Parameter	Estimate	Std. Error	z-statistic	*p*-value
Intercept	−0.383	0.345	−1.109	0.268
ORDER (SO vs. OS)	6.196	0.722	8.582	<0.0001
CONSTRCUCTION TYPE (trans vs. rel)	1.363	0.383	3.557	0.0004
NP (pro vs. N)	−1.958	0.450	−4.350	<0.0001
CONSTRUCTION TYPE:NP	1.724	0.593	2.904	0.004

In order to correctly interpret the object-first sentences, children had to use case marking without support from word order (see examples in Table 5). As indicated by the main effect for construction type (see Table 8), the older children made more use of case marking without support from any other cues in their interpretation of simple transitives. Their performance on the object-first simple transitives with nominative demonstrative pronouns did not differ from their performance on the object-first simple transitives with nominative lexical NPs (compare trans OPro and trans OS in Figure 2). Looking at the object-first RCs (rel OS and rel OPro), we even see a negative effect for the pronoun. The six-year-olds performed worse on the object-first RCs with nominative demonstrative pronouns than on the object-first RCs with nominative lexical NPs.

**Table 8.** GLMM main effects and 2-way interactions 6-year-olds Experiment 2. Correct interpretation of simple transitives and transitive relative clauses with case marking on lexical NPs and demonstrative pronouns. Object-first sentences only.^8^

Parameter	Estimate	Std. Error	z-statistic	*p*-value
Intercept	−0.376	0.374	−1.005	0.315
CONSTRUCTION TYPE (trans vs. rel)	1.381	0.396	3.489	0.0005
NP (pro vs. N)	−2.123	0.480	−4.424	<0.0001
CONSTRUCTION TYPE:NP	1.873	0.624	3.001	0.003

**Table 9:** GLMM main effects 3-year-olds Experiment 2. Correct interpretation of simple transitives and transitive relative clauses with case marking on lexical NPs and demonstrative pronouns.^9^

Parameter	Estimate	Std. Error	z-statistic	*p*-value
Intercept	−0.086	0.137	−0.624	0.532
ORDER (SO vs. OS)	0.766	0.173	4.441	<0.0001

The model for the 3-year-olds children showed a main effect for order only (see Table 9). The 3-year-olds only made use of case marking when this cue was supported by word order. As can be seen in Figure 2, they performed better on the subject-first sentences than on the object-first sentences. When asked to interpret object-first sentences, in which case marking is in conflict with word order, the younger children performed at chance. In other words, as a group, the three-year-olds did not show a preference for either cue. When we look at individual children’s performance, in each condition, there are 3 to 6 children who consistently followed word order and 9 to 12 children who mainly followed word order. The numbers vary because children do not necessarily show the same tendencies across conditions. For example, one child interpreted the vast majority of object-first sentences as subject-first, whereas another child interpreted all of the trans OS sentences as subject-first and all of the trans OPro sentences as object-first. Similar patterns were found when we looked at individual children in the older age group.

Finally, whether the 3-year-olds were asked to interpret a simple transitive or a transitive RC did not affect their performance. Similarly, whether the second NP or the embedded NP was expressed by a demonstrative pronoun or a lexical NP did not affect the younger children’s performance.

### Discussion experiment 2

These results support the assumption that older children are more sensitive to cue reliabilities and validities than younger children (MacWhinney, 2007; McDonald, 1986). The 3-year-olds did not follow the more reliable case-marking cue and ignored the less reliable word-order cue. When asked to interpret conflict sentences where word order points to an SO interpretation and case marking points to an OS interpretation, they followed word order and case marking equally often (see Figure 2). Even though some children consistently followed word order in some conditions, most of them did not show any clear patterns across conditions.

The 6-year-olds, on the other hand, followed the more reliable cue of case marking and mostly ignored word order in their interpretation of simple transitives. If their sensitivity to cue reliabilities and validities were construction-specific, the older children should have also been more likely to follow case marking and ignore word order in their interpretation of transitive RCs than in their interpretation of simple transitives. However, our results show the reverse pattern. That the older children did not reliably follow case in their interpretation of object-first RCs could be caused by the fact that the case-marking cue is provided late in the sentence and is thus difficult to integrate in the online processing (Choi & Trueswell, 2010).

The object-first interpretation of simple transitives is signaled on the very first word; i.e., the determiner preceding the noun it refers to:

1. **Den**  **Vogel** schubst jetzt der Hase.
**the-ACC** **bird** pushes  now the-NOM rabbit
  ‘the rabbit is pushing the bird now’

The object-first interpretation of transitive RCs, on the other hand, is only signaled on the relative pronoun following the noun it refers to. In addition, it can be in conflict with the determiner preceding the same noun, which means that there is not only a conflict between word order and case marking, but also a conflict within case marking:

1. **Der   Vogel, den**   der   Hase schubst.
**the-NOM** **bird** **that-ACC** the-NOM rabbit pushes 
  ‘the bird that the rabbit is pushing’

In order to avoid this conflict, in Experiment 3, we also tested children with object-first RCs where the head NP plays the object role in both the main clause and in the relative clause. In other words, in a sentence like (11), both the determiner preceding the head NP (*Esel* ‘donkey’) and the relative pronoun following the head NP are marked accusative.

1. Zeig mal **den**  **Esel**,  **den**   der   Vogel schubst.
  Show PRT **the-ACC donkey** **that-ACC** the-NOM bird pushes 
  ‘show (me) the donkey that the bird is pushing’

Finally, if children’s sensitivity to cue reliabilities and validities were item-specific, children from both age groups should have also been better at processing case-marked pronouns than case-marked lexical NPs. This is not supported by our results. This might be due to the same reason mentioned above; the case-marked pronouns appear late in the sentence.

However, this cannot really explain why we even found a negative pronoun effect in the older children’s interpretation of object-first RCs. When asked to interpret transitive RCs with a demonstrative pronoun in the subject slot, such as *der Vogel, den **der** jetzt schubst* ‘the-NOM bird that-ACC **he-NOM** is pushing now,’ German-speaking three-year-olds performed at chance, whereas the 6-year-olds showed a floor effect (see Figure 2). In other words, the older children were consistently wrong. This finding is similar to what Booth, MacWhinney, and Harasaki (2000) have reported in their paper on the influence of short-term and working memory on children’s processing of RCs. More specifically, Booth et al. (2000) tested the comprehension of complex sentences with RCs in children between the age of 8;0 and 11;0. In their second experiment, they found that high digit-span children were more consistent in their application of an incorrect local attachment strategy than low digit-span children. After hearing a complex sentence such as *the man that the captain invited built the stage for the band*, for example, the high digit-span children mostly confirmed an incorrect statement such as *the captain built the stage*, whereas the low digit-span children showed more random behavior.

The consistent misinterpretation that we also found in our 6-year olds might suggest that when they hear a noun phrase with nominative case marking (*der Vogel* ‘the-NOM bird‘ in example (10) above), they interpret it as agent. Some children stick to that interpretation. Other children change this initial interpretation: When another lexical NP with nominative case marking comes up (*der Hase* ‘the-NOM rabbit‘ in example (10) above), they interpret this as agent and arrive at a correct interpretation. This latter behavior could also be driven by a local-attachment strategy: the children only pay attention to the final NP and VP (… ***der Hase*** *schubst* ‘… **the-NOM rabbit** is pushing’). However, this does not occur with case-marked pronouns in the subject slot because this would cause additional processing costs; that is, children would first need to retrieve the correct antecedent for the pronoun. Moreover, the demonstrative pronouns might not be salient enough to provoke a reanalysis.

Overall, demonstrative pronouns are more frequent than personal pronouns. However, our corpus study has shown that the vast majority of object-first RCs contain a personal pronoun in the subject slot (e.g., *das Fleisch, was **ich** von der Schweinshaxe abknabbere* ‘the meat that **I** nibble off the knuckle of pork’). Moreover, most of these personal pronouns are first- or second-person pronouns. Therefore, in Experiment 3, we also tested whether 4- and 6-year-old children are able to correctly interpret object and subject RCs with first-person personal pronouns in the subject or object slot.

## Experiment 3: Transitive relative clauses with case marking on lexical NPs and personal pronouns

In this final experiment we only tested children’s comprehension of transitive RCs. The two main questions were (1) whether children could make use of case marking in transitive RCs when it is marked on first-person personal pronouns and (2) whether children could make use of case marking in transitive RCs when it is not in conflict with case marking in the main clause.

### Participants

We tested two age groups: 24 monolingual German 4-year-olds (mean = 4;3, range: 4;0-4;5) and 24 monolingual 6-year-olds (mean = 6;10, range: 6;8-6;11) were included in the study. The participants in the younger age group were older than in Experiments 1 and 2 because Experiment 3 was more demanding. Children were tested on transitive RCs only. Another five children were tested but were excluded from the main analysis due to technical problems (3), only ambiguous responses (1 always pointed to both movies), or fussiness (1). The 4-year-olds were recruited from nurseries in a midsize German city and tested in a quiet room in their nurseries. The 6-year-olds were recruited from primary schools in the same city and were tested in a quiet room in their schools during after-school activities. None of the children had any known language impairments.

### Materials

Twenty-four test sentences were constructed. The first manipulation was word order (SO versus OS). The second manipulation was consistency of case marking. In half of the test sentences the head NP played the same syntactic role in the main clause as in the RC. So the case marking on the determiner preceding the head NP was the same as the case marking on the relative pronoun following the head NP (nominative for subject-first RCs and accusative for object-first RCs). For the other half of the test sentences, the head NP played different roles in the main clause and in the RC and there was conflict between the case marking on the determiner preceding the head NP and the relative pronoun following the head NP. The third manipulation was type of NP. The embedded NP was either expressed by a lexical NP or by a first-person personal pronoun. These manipulations resulted in eight conditions (see Table 10) with three items in each condition.

**Table 10.** Conditions and example sentences for transitive RCs with case on lexical NPs and personal pronouns.

con SO	Wo ist der Hase, der den Vogel schubst? ‘where is the-NOM rabbit who-NOM the-ACC bird pushes’
incon SO	Zeig mal den Hasen, der den Vogel schubst! ‘show (me) the-ACC rabbit who-NOM the-ACC bird pushes’
con SPro	Wo ist der Hase, der mich schubst? ‘where is the-NOM rabbit who-NOM me-ACC pushes’
incon SPro	Zeig mal den Hasen, der mich schubst! ‘show (me) the-ACC rabbit who-NOM me-ACC pushes’
con OS	Zeig mal den Vogel, den der Hase schubst! ‘show (me) the-ACC bird who-ACC the-NOM rabbit pushes’
incon OS	Wo ist der Vogel, den der Hase schubst? ‘where is the-NOM bird who-ACC the-NOM rabbit pushes’
con OPro	Zeig mal den Vogel, den ich schubse! ‘show (me) the-ACC bird who-ACC I-NOM push’
incon OPro	Wo ist der Vogel, den ich schubse? ‘where is the-NOM bird who-ACC I-NOM push’

‘Con’ stands for test sentences with consistent case marking, ‘incon’ stands for test sentences without consistent case marking. ‘SO’ stands for subject-first RCs with two lexical NPs, ‘SPro’ stands for subject-first RCs where the embedded NP, i.e., the object, is expressed by a first-person personal pronoun, ‘OS’ stands for object-first RCs with two lexical NPs, and ‘OPro’ stands for object-first RCs where the embedded NP; that is, the subject, is expressed by a first-person personal pronoun.

As in Experiments 1 and 2, the RCs were all right-branching, that is, not center-embedded. They were attached to main clauses, such as *show me the X* or *where is the X*. To ensure clear case marking, the animal names used in the test sentences have masculine gender in German. The animals were the same as in Experiment 2: rabbit, monkey, tiger, bear, lion, dog, elephant, frog, donkey, tomcat, hedgehog, and bird. The actions were the same as in Experiments 1 and 2: feed, push, comb, tickle, stroke, and wash. And we used the same movie pairs as in Experiment 2.

Eight experimental orders were created by randomly matching conditions with movie pairs. Three children of each age group were tested with each list. The movies played on the left and right side of a computer screen (25.5cm x 41cm). Within each order, the target, that is, the movie described by the test sentence, appeared on both sides equally often (12 times on the right and 12 times on the left). The same side was never the correct choice more than twice in a row. In none of the experimental orders did the correct choice alternate regularly (e.g., LRLRLRLR). For each movie pair we counterbalanced between subjects whether the target movie played on the left or right side of the screen.

### Procedure

The procedure was similar to Experiments 1 and 2. However, since the children in Experiment 3 were 4;0 and older, we left out the extra labeling phase before showing the movies and combined the labeling with the familiarization phase. So, at the beginning of each test session, children saw a sample of six movie pairs displaying all 12 animals and 6 actions. In the familiarization phase, the movies from each pair were played after one another and each movie was played for 10 seconds (e.g., tiger feeding bear followed by bear feeding tiger). While the children were watching the movies, they were asked to label the animals and the actions displayed.

Then the test trials were presented in one block. In order to allow for consistent and inconsistent case marking, the test sentences were embedded in linguistic contexts that were different from the ones used in Experiments 1 and 2. Depending on whether the condition involved consistent or inconsistent case marking and whether the test sentence was a subject-first or object-first RC, the test question started with *wo ist der X* ‘where is the-NOM X’ or *zeig mal den X* ‘show (me) the-ACC X’ (see Table 10). All test sentences were pre-recorded.

Before each test trial the experimenter (re-) introduced a hand puppet. For the test sentences containing a personal pronoun it was the referent of the pronoun (i.e., *ich* ‘I-NOM’ or *mich* ‘me-ACC’). For the test sentences containing only lexical NPs, it was a random puppet that did not occur in the movies and the test sentence. The experimenter then said, for example, *lass uns mal gucken wen der Vogel sucht* ‘let’s see who-ACC the-NOM bird is looking for’ (the bird being the puppet that has just been (re-) introduced). The hand puppet was placed next to the speaker that would play the test sentence and the experimenter asked the puppet, for example, *Vogel, wen suchst Du* ‘bird, who-ACC are you looking for.’ After a couple of trials, this question was often asked by the children. Then the movies were played together with the test sentence (e.g., *zeig mal den Bär, den ich schubse* ‘show (me) the-ACC bear who-ACC I-NOM push’). As in Experiments 1 and 2, the movies played for ten seconds and the test sentence was played twice. The children pointed to one of the two movies while they were playing. When the children did not point, the trial was repeated once.

### Results experiment 3

**Figure 3.**
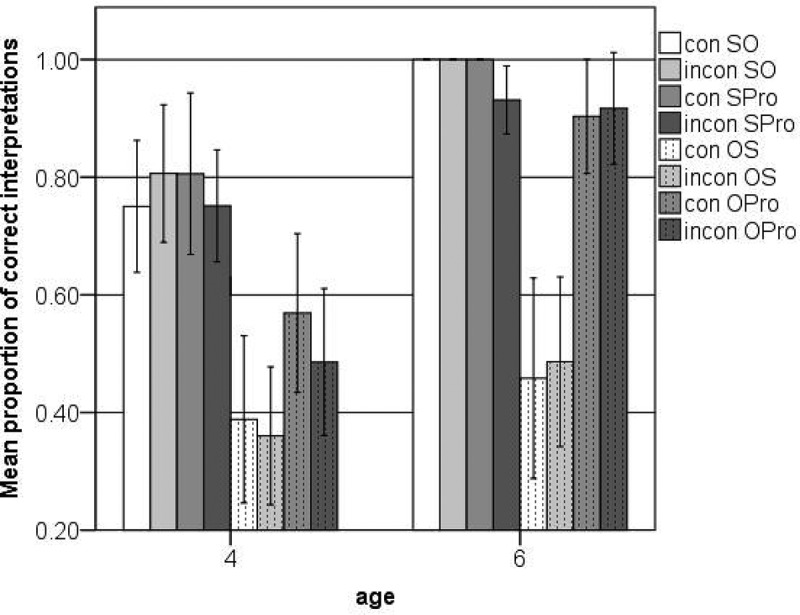
Correct interpretations of transitive relative clauses with case marking on lexical NPs (SO, OS) and personal pronouns (SPro, OPro), consistent or inconsistent with the case marking in the clause (con vs. incon) (Experiment 3)

Figure 3 shows the percentage of correct interpretations of all trials in each condition. The GLMM analysis procedure was the same as for Experiments 1 and 2. First we analyzed the data from both age groups together. The final model showed significant main effects for order (SO vs. OS), form of NP (lexical NP vs. personal pronoun), and age (4- vs. 6-year-olds), as well as interactions between order and form of NP, order and age, and form of NP and age (see Table 11).

**Table 11.** GLMM main effects and 2-way interactions Experiment 3. Correct interpretations of transitive RCs with case on lexical NPs and personal pronouns.^10^

Parameter	Estimate	Std. Error	z-statistic	*p*-value
Intercept	−0.712	0.266	−2.673	0.007
ORDER (SO vs. OS)	2.288	0.290	7.900	<0.0001
NP (pro vs. N)	0.949	0.262	3.627	0.0003
AGE (6 vs. 3)	0.701	0.360	1.950	0.051
ORDER:NP	−1.209	0.383	−3.158	0.002
ORDER:AGE	1.832	0.567	3.233	0.001
NP:AGE	1.484	0.401	3.699	0.0002

Next we did separate analyses for the 4- and 6-year-olds. After removing all nonsignificant effects and two-way interactions, the final model for the 4-year-olds showed main effects for order and form of NP (see Table 12). As Figure 3 suggests, the younger children found it easier to correctly interpret the subject-first RCs than the object-first RCs. Even though the GLMM did not show a significant interaction between order and type of NP (*p* = .131), Figure 3 also suggests that the type of NP only influenced the 4-year-olds’ interpretation of object-first RCs. In order to further investigate this, we did separate analyses for the subject-first and object-first RCs. For the subject-first RCs, this showed no significant effects or interactions. For the object-first RCs, the final model showed a main effect for type of NP (see Table 13), confirming that the younger children found it easier to interpret object-first RCs with a personal pronoun in the subject slot than object-first RCs with a lexical NP in the subject slot.

**Table 12.** GLMM main effects 4-year-olds Experiment 3. Correct interpretations of transitive RCs with case on lexical NPs and personal pronouns.^11^

Parameter	Estimate	Std. Error	z-statistic	*p*-value
Intercept	−0.434	0.224	−1.940	0.052
ORDER (SO vs. OS)	1.652	0.202	8.163	<0.0001
NP (pro vs. N)	0.418	0.207	2.018	0.044

**Table 13.** GLMM main effects 4-year-olds Experiment 3. Correct interpretations of transitive RCs with case on lexical NPs and personal pronouns. Object-first sentences only.^12^

Parameter	Estimate	Std. Error	z-statistic	*p*-value
Intercept	−0.531	0.192	−2.769	0.006
NP (pro vs. N)	−0.647	0.242	2.667	0.007

After removing all nonsignificant effects and two-way interactions, the final model for the 6-year-olds showed main effects for order and type of NP (see Table 14). Like the younger children, they found it easier to interpret subject-first RCs than object-first RCs (see Figure 3). Figure 3 also suggests that the form of NP only had an effect on the older children’s interpretation of the object-first RCs. We were unable to fit a model including interactions with order due to zero counts in some categories (i.e., some 6-year-olds consistently misinterpreted all items in one or several object-first conditions as subject-first). In addition, due to ceiling effects it was impossible to fit a model for the subject-first RCs only. However, a separate model for the object-first RCs confirmed that the 6-year-olds found it much easier to interpret object-first RCs with a personal pronoun in the subject slot than object-first RCs with a lexical NP in the subject slot (see Table 15).

**Table 14.** GLMM main effects 6-year-olds Experiment 3. Correct interpretations of transitive RCs with case on lexical NPs and personal pronouns.^13^

Parameter	Estimate	Std. Error	z-statistic	*p*-value
Intercept	0.023	0.313	0.073	0.942
ORDER (SO vs. OS)	4.080	0.543	7.519	<0.0001
NP (pro vs. N)	2.454	0.343	7.159	<0.0001

**Table 15.** GLMM main effects 6-year-olds Experiment 3. Correct interpretations of transitive RCs with case on lexical NPs and personal pronouns. Object-first sentences only.^14^

Parameter	Estimate	Std. Error	z-statistic	*p*-value
Intercept	−0.201	0.408	−0.492	0.623
NP (pro vs. N)	3.497	0.461	7.478	<0.0001

None of the models showed any effect for or interaction with consistency. That is, whether the case marking on the determiner preceding the head NP was consistent or inconsistent with the case marking on the relative pronoun following the head NP had no effect on children’s interpretation of subject- or object-first RCs. In other words, whether the head NP played the same syntactic role in the main clause as in the RC did not influence children’s interpretation of transitive RCs.

### Discussion experiment 3

In Experiment 3 we investigated two issues: (1) Can children make use of (nominative) case marking in transitive RCs when it is marked on first-person personal pronouns (as opposed to lexical NPs)? (2) Can children make use of case marking in transitive RCs when it is not in conflict with case marking in the main clause? The answer to the first question is yes. Both 4- and 6-year-old children were more likely to correctly interpret object-first RCs with first-person personal pronouns in the subject slot (e.g., ‘where is the-NOM bird that-ACC **I-NOM** push’) than object-first RCs with lexical NPs in the subject slot (e.g., ‘where is the-NOM bird that-ACC **the-NOM rabbit** pushes’). The older children were almost at ceiling in the conditions with first-person pronominal subjects, whereas their performance in the conditions with lexical-NP subjects was at chance (see Figure 3).

The answer to the second question is no. The case marking on the determiner preceding the head NP had no influence on the interpretation of the case marking on the relative pronoun following the head NP or the interpretation of the case marking on the embedded NP. In other words, whether or not the head NP played the same syntactic role in the main clause as in the RC had no influence on children’s comprehension of the object-first (or subject-first) RCs. Moreover, our results suggest that children actually make better use of the form of the very last NP than of the form of the case marked relative pronoun. Even, though the relative pronoun occurs before the embedded NP, children do not make reliable use of the case marking on the relative pronoun. This has also been demonstrated in Experiment 2. They do, however, make use of case marking on the embedded NP when it comes in the form of a first-person personal pronoun.

Even though we did not measure children’s memory spans, these results support the assumption that children’s use of cues is not just constrained by memory (Arosio et al., 2012) and that they are, in fact, able to make use of cues even if they appear late in the sentences (Choi & Truswell, 2010). Our findings suggest that the timing and positioning of cues interact with other factors such as lexical specificity and saliency. Children can make use of cues that appear late in the sentence if the cue comes in a specific form (e.g., in the form of a first-person personal pronoun). As discussed above, case marking on personal pronoun is also more salient than case marking on determiners or demonstrative pronouns.

## General discussion

### Reliability of word order and case across age and constructions

In Experiment 1, the younger children were more likely to follow word order in their interpretation of simple transitives than in their interpretation of transitive RCs. This supports our suggestion that children’s use of cues might initially be construction-specific because, as our corpus study suggests, word order in simple transitives is much more reliable than word order in transitive RCs. Alternatively, we could say that most simple transitives are S(V)O, whereas most transitive RCs are actually OS(V). The older children, on the other hand, also followed word order in their interpretation of transitive RCs (i.e., they interpreted the transitive RCs as SO(V)), which might suggest that they use syntactic cues on a more global, construction-general level. However, this global, construction-general use of cues was not evident in the older children’s use of case marking.

In Experiment 2, the 6-year-olds made use of case marking to interpret simple transitives with an object-first word order. Unlike the 3-year-olds, the older children started to follow the more reliable case-marking cue, but they did not reliably make use of case marking to interpret transitive RCs with an object-first word order. The findings from Experiment 3 suggest that this is due to the fact that children’s use of cues may not just be construction-specific, but that this construction specificity also interacts with lexical specificity and saliency of specific cues.

In addition, we cannot fully rule out other formal factors that are confounded with construction specificity. Our transitive RCs and simple-transitive test items were different in a number of ways. As has been pointed out by one of the anonymous reviewers, the simple transitives in Experiments 1 and 2 contained extra words, such as *jetzt* and *mal* ‘now,’ which we inserted to make the simple transitives as long as the transitive RCs. Moreover, the timing and positioning of cues differ across RCs and simple transitives. In Experiment 3, we have investigated the issue of timing and positioning of cues in transitive RCs. However, we have not yet explored this issue in simple transitives. In order to find clearer evidence for children’s construction-specific processing and use of morpho-syntactic cues, one could, for example, also compare children’s interpretation of embedded simple transitives with verb-final word order, such as *ich sehe, dass das Pferd den Hund jagt* ‘I see that that the-NOM/ACC horse is chasing the-ACC dog’ and their interpretation of transitive RCs, such as *da ist das Pferd, das den Hund jagt* ‘there’s the-NOM/ACC horse that-NOM/ACC is chasing the-ACC dog.’ Then the timing and position of case-marked items would be more similar across constructions.

### Lexical specificity and saliency of case marking

In Experiment 3, the older children were able to use case marking and correctly interpret most object-first RCs when case was marked on the first-person pronoun *ich* ‘I-NOM.’ The younger children were also more likely to comprehend object-first RCs with a first-person pronoun in the subject slot (e.g., ‘where is the-NOM bird that-ACC **I-NOM** push’) than object-first RCs with a lexical NP in the subject slot (e.g., ‘where is the-NOM bird that-ACC **the-NOM rabbit** pushes’). In Experiment 2, case was marked on lexical NPs, on relative pronouns, and/ or on third-person demonstrative pronouns, and children from either age group did not make reliable use of it in their interpretation of object-first RCs. That children struggled with case marking on lexical NPs is probably due to the fact that (nominative) case is most often marked on pronouns (Dittmar et al., 2008). That children also struggled with case marking on relative and demonstrative pronouns is probably due to the fact that in transitive RCs, nominative case is most often marked on personal pronouns. Moreover, case marking on relative pronouns, demonstrative pronouns and lexical NPs is less salient than case marking on personal pronouns.

In addition, we used third-person demonstrative pronouns in Experiment 2 and first-person personal pronouns in Experiment 3. First-person personal pronouns are not just more salient than third-person demonstratives. It is also easier to retrieve and process the referents of first-person pronouns because the referents of first- and second-person pronouns are naturally given and thus highly accessible (e.g., Chafe, 1994; Warren & Gibson, 2002). Similarly, it has been suggested that object-first RCs containing first- or second-person pronouns in the subject slot are easier to process than object-first RCs with third-person pronouns or lexical NPs in the subject slot because it is easier to link referring expressions to syntactic roles when the referring expressions differ in their form (e.g., Gordon, Hendrick, & Johnson, 2001). In other words, figuring out syntactic relations in a sentence containing *the dog* and *I* is easier than determining syntactic relations in a sentence containing *the dog* and *it* or *the dog* and *the lion*.

### Form or function?

Pronouns and lexical NPs do not just differ in their form. They also tend to have different functions. Pronouns are more likely to refer to given discourse referents and function as subjects than are lexical NPs (Du Bois, 1987). That pronouns tend to encode given discourse referents was also reflected in our procedure where we (re-) introduced the referent of the pronoun before we presented a test sentence containing a pronoun. Thus, both in spontaneous speech and in our experiments the form and function of pronouns was confounded. However, a comparison of the results from Experiments 2 and 3 suggests that the form of the pronoun plays a unique role in children’s processing of object-first RCs. In both Experiments 2 and 3, the pronoun referred to a given discourse entity, but in Experiment 2 we used the third-person demonstrative pronoun *der* ‘he-NOM,’ whereas in Experiment 3, we used the first-person personal pronoun *ich* ‘I.’ It turned out that only first-person personal pronouns lead to better processing of object-first RCs. This finding suggests that children’s processing of RCs is constrained by lexical specificity rather than discourse function (for a similar account concerning adults’ processing of object RCs see Reali & Christiansen, 2007). However, we need to acknowledge again that the case marking on personal pronouns is also more salient than the case marking on demonstrative pronouns and that we used third-person demonstrative pronouns in Experiment 2 and first-person personal pronouns in Experiment 3. Therefore, future research should have a closer look at the interaction between form and function of pronominal forms and lexical NPs in children’s sentence processing.

### Position and timing of case marking

We also speculated whether the use of case marking in transitive RCs is more difficult than the use of case marking in simple transitives because in transitive RCs the head NP can play different roles in the main clause and in the RC, which can lead to conflicting information within case marking (see also MacWhinney & Pleh, 1988). In Experiment 3, the case marking on the determiner preceding the head NP was either consistent or inconsistent with the case marking on the relative pronoun following the head NP. It turned out that the consistency of the case marking had no effect on children’s interpretation of object-first or subject-first RCs (see Figure 3). In other words, whether or not the determiner preceding the head NP was marked nominative or accusative had no effect on children’s interpretation of the case marking provided on the relative pronoun or the case marking on the embedded NP following the head NP.

The only factor that had a significant effect on children’s interpretation of object-first RCs was whether the embedded NP came in the form of a nominative lexical NP or in the form of a nominative first-person personal pronoun. The older children performed almost at ceiling on object-first RCs with first-person personal pronouns in the subject slot. When these were marked nominative, the older children had almost no problems (re-) interpreting transitive RCs as object-first. Similar trends were found for the younger children. These findings are interesting because they suggest that both 4- and 6-year-old children are able to (re-) interpret sentences as object-first and that they can use cues that appear late in the sentence to guide this (re-) interpretation.

As has been suggested by Guasti et al. (2012), it is not necessarily the position of the cue that determines whether children (and adults) can make use of it in their online processing. It might also be the type of cue. Remember that Guasti et al. found that Greek-speaking children are more likely to use case marking than agreement in their interpretation of object-first RCs even though the agreement information on the embedded verb is encountered before the case-marking information on the embedded NP. Unfortunately, Guasti et al. do not discuss whether case marking is also more available and/ or reliable than agreement in Greek. But together with the findings from the current study, this suggests that local cues, such as case marking (Slobin, 1982), can be used even when they occur late in the sentence. In other words, global cues like agreement need to be presented early in the sentence, but local cues like case marking can also be used by children when they appear late in the sentence, given that they come in a specific form. This might also explain why the children in Choi and Truswell’s (2010) study seemed to be unable to revise their initial parsing decision based on a cue that was presented late in the sentence. They tested children’s use of a more global cue by investigating whether they make use of lexical information on the sentence-final verb in order to determine whether sentence-initial prepositional phrases need to be (re-) interpreted as verb arguments (as in (12)) or as noun modifiers (as in (13)).

1. *Naypkhin-**ey**  kaykwuli-lul  cipu-sey-yo*.
  napkin-**GEN**  frog-ACC   pick up
  ‘Pick up the frog on the napkin’
2. *Naypkhin-**ey**  kaykwuli-lul  nohu-sey-yo*.
  napkin-**LOC**  frog-ACC   put
  ‘Put the frog on the napkin’

Unlike Choi and Trueswell (2010), we did not measure children’s eye movements. Furthermore, Choi and Trueswell did not measure children’s executive-control abilities to determine correlations between this ability and the likelihood of revising a sentence interpretation based on cues appearing late in the sentence. Finally, as suggested by Arosio et al.’s (2012) findings, whether children use specific cues might also depend on their short-term memories. Future research involving eye tracking will have to integrate all these factors and methods in order to investigate how children’s ability to (re-) interpret sentences interacts with the type of cue (local vs. global), the position/timing of the cue (sentence-initial or –final), short-term memory, and executive function.

## Conclusion

This study is one of the first that compares children’s use of syntactic cues across different constructions and lexical items. Our results suggest that especially younger children use word order and case marking on a construction-specific level. In addition, children from both age groups are more likely to pay attention to (nominative) case on personal pronouns than (nominative) case on lexical NPs or demonstrative pronouns. Finally, our findings suggest that there is an interaction between type of cues, form of cues, and timing of cues. Provided that they come in a specific, salient, form, local cues like case marking can be integrated in children’s sentence processing even if they are encountered late in the sentence.

## Notes

1. In certain phonetic contexts (e.g., after schwas) case endings also appear on adjectives and nouns.

2. We would like to thank Heike Behrens for making these transcripts available to us.

3. As in previous studies, validity is the product of availability (how often the cue is present in transitive RCs) x reliability (how often the cue points to the correct interpretation when it is available).

4. In the models fitted there was either only a subject level random intercept or independent subject level and item level random intercepts. The data set would need a greater number of subjects and a greater number of observations per subject to justify random slopes. The predictor variables were coded as 0 and 1 (e.g., age 6 = 0; age 3 = 1).

5. Subject random effect variance: 0.714; -2 x LogLikelihood = 773.97

6. Subject random effect variance: 0.325; -2 x LogLikelihood = 582.177

7. Subject random effect variance: 1.188; -2 x LogLikelihood = 338.58

8. Subject random effect variance: 1.587; -2 x LogLikelihood = 303.5

9. Subject random effect variance: 0.115; -2 x LogLikelihood = 764.79

10. Subject random effect variance: 0.791; -2 x LogLikelihood = 994.90

11. Subject random effect variance: 0.415; -2 x LogLikelihood = 676.91

12. Subject random effect variance: 0.154; -2 x LogLikelihood = 388.06

13. Subject random effect variance: 0.415; -2 x LogLikelihood = 322.6

14. Subject random effect variance: 3.002; -2 x LogLikelihood = 243.59

## References

[CIT0001] Altmann G., Kamide Y. (1999). *Incremental interpretation at verbs: Restricting the domain of subsequent reference*.

[CIT0002] Arnon I. (2010). Rethinking child difficulty: The effect of NP type on children’s processing of relative clauses in Hebrew. *Journal of Child Language*.

[CIT0003] Arosio F., Guasti M. T., Stucchi N. (2011). Disambiguating information and memory resources in children’s processing of Italian relative clauses. *Journal of Psycholinguistics Research*.

[CIT0004] Arosio F., Yatsushiro K., Forgiarini M., Guasti M. T. (2012). Morphological information and memory resources in children’s processing of relative clauses in German. *Language Learning and Development*.

[CIT0005] Baayen R. H., Davidson D. J., Bates D. M. (2008). Mixed-effects modeling with crossed random effects for subjects and items. *Journal of Memory and Language*.

[CIT0006] Bates D. M., Maechler M. (2010). lme4: Linear mixed-effects models using S4 classes. R package version.

[CIT0007] Bates E., Devescovi A., D’Amico S. (1999). Processing complex sentences: A cross-linguistic study. *Language and Cognitive Processes*.

[CIT0008] Bates E., MacWhinney B., MacWhinney B. (1987). Competition, variation, and language learning. *Mechanisms of language aquisition*.

[CIT0009] Bates E., MacWhinney B., MacWhinney B., Bates E. (1989). Functionalism and the competition model. *The cross-linguistic study of sentence processing*.

[CIT0010] Bates E., MacWhinney B., Caselli C., Devescovi A., Natale F., Venza V. (1984). A cross-linguistic study of the development of sentence interpretation strategies. *Child Development*.

[CIT0011] Behrens H. (2006). The input-output relationship in first language acquisition. *Language and Cognitive Processes*.

[CIT0012] Booth J. R., MacWhinney B., Harasaki Y. (2000). Developmental differences in visual and auditory processing of complex sentences. *Child Development*.

[CIT0013] Brandt S., Diessel H., Tomasello M. (2008). The acquisition of German relative clauses: A case study. *Journal of Child Language*.

[CIT0014] Brandt S., Kidd E., Lieven E., Tomasello M. (2009). The discourse bases of relativization: An investigation of young German and English-speaking children’s comprehension of relative clauses. *Cognitive Linguistics*.

[CIT0015] Chafe W. *Discourse, Consciousness, and time: The flow and displacement of conscious experience in speaking and writing*.

[CIT0016] Chan A., Lieven E., Tomasello M. (2009). Children’s understanding of the agent-patient relations in the transitive construction: Cross-linguistic comparisons between Cantonese, German and English. *Cognitive Linguistics*.

[CIT0017] Choi Y., Trueswell J. (2010). Children’s (in)ability to recover from garden paths in a verb-final language: Evidence for developing control in sentence processing. *Journal of Experimental Child Psychology*.

[CIT0018] Clahsen H. (1984). Der Erwerb von Kasusmarkierungen in der deutschen Kindersprache. *Linguistische Berichte*.

[CIT0019] Devescovi A., D’Amico S., Gentile P. (1999). The development of sentence comprehension in Italian: A reaction time study. *First Language*.

[CIT0020] Dittmar M., Abbot-Smith K., Lieven E., Tomasello M. (2008). German children’s comprehension of word order and case marking in causative sentences. *Child Development*.

[CIT0021] Du Bois J. (1987). The discourse basis of ergativity. *Language*.

[CIT0022] Frazier L., Clifton C. (1989). Successive cyclicity in the grammar and the parser. *Language and Cognitive Processes*.

[CIT0023] Friederici A. D., Steinhauer K., Mecklinger A., Meyer M. (1998). Working memory constraints on syntactic ambiguity resolution as revealed by electrical brain responses. *Biological Psychology*.

[CIT0024] Gordon P.C., Hendrick R., Johnson M. (2001). Memory interference during language processing. *Journal of Experimental Psychology: Learning, Memory and Cognition*.

[CIT0025] Guasti M.T., Stavrakaki S., Arosio F. (2012). Cross-linguistic differences and similarities in the acquisition of relative clauses: Evidence from Greek and Italian. *Lingua*.

[CIT0026] Grünloh T., Lieven E., Tomasello M. (2011). German children use prosody to identify participant roles in transitive sentences. *Cognitive Linguistics*.

[CIT0027] Jaeger T. F. (2008). Categorical data analysis: Away from ANOVAs (transformation or not) and towards logit mixed models. *Journal of Memory and Language*.

[CIT0028] Kempe V., MacWhinney B. (1998). The acquisition of case-marking by adult learners of Russian and German. *Studies in Second Language Acquisition*.

[CIT0029] Kempe V., MacWhinney B. (1999). Processing of morphological and semantic cues in Russian and German. *Language and Cognitive Processes*.

[CIT0030] Kidd E., Brandt S., Lieven E., Tomasello M. (2007). Object relatives made easy: A cross-linguistic comparison of the constraints influencing young children’s processing of relative clauses. *Language and Cognitive Processes*.

[CIT0031] Kidd E., Stewart A., Serratrice L. (2011). Children do not overcome lexical biases where adults do: The role of the referential scene in garden-path recovery. *Journal of Child Language*.

[CIT0032] MacDonald M. C., MacWhinney B. (1999). Distributional information in language comprehension, production, and acquisition: Three puzzles and a moral. *The emergence of language* (pp. 177–196).

[CIT0033] MacWhinney B. (2000). *The CHILDES-project: Tools for analyzing talk*.

[CIT0034] MacWhinney B., Ellis N., Robinson P. (2007). A unified model. *Handbook of cognitive linguistics and second language acquisition*.

[CIT0035] MacWhinney B., Bates E. (1989). *The cross-linguistic study of sentence processing*.

[CIT0036] MacWhinney B., Bates E., Kliegl R. (1984). Cue validity and sentence interpretation in English, German, and Italian. *Journal of Verbal Learning & Verbal Behavior*.

[CIT0037] MacWhinney B., Pleh C. (1988). The processing of restrictive relative clauses in Hungarian. *Cognition*.

[CIT0038] MacWhinney B., Pleh C., Bates E. (1985). The development in sentence interpretation in Hungarian. *Cognitive Psychology*.

[CIT0039] McDonald J. L. (1986). The development of sentence comprehension strategies in English and Dutch. *Journal of Experimental Child Psychology*.

[CIT0040] Nitschke S., Kidd E., Serratrice L. (2010). First language transfer and long-term structural priming in comprehension. *Language and Cognitive Processes*.

[CIT0041] Pine J. M., Lieven E. V. M., Rowland C. F. (1998). Comparing different models of the development of the English verb category. *Linguistics*.

[CIT0042] Reali F., Christiansen M.H. (2007). Word-chunk frequencies affect the processing of pronominal object-relative clauses. *Quarterly Journal of Experimental Psychology*.

[CIT0043] Seidenberg M. S., MacDonald M. C. (1999). A probabilistic constraints approach to language acquisition and processing. *Cognitive Science*.

[CIT0044] Slobin D. I., Wanner E., Gleitman L. (1982). Universal and particular in the acquisition of language. *Language acquisition: The state of the art*.

[CIT0045] Stoll S., Abbot-Smith K., Lieven E. (2009). Lexically restricted utterances in Russian, German and English child directed speech. *Cognitive Science*.

[CIT0046] Suzuki T. (2011). A case-marking cue for filler–gap dependencies in children’s relative clauses in Japanese. *Journal of Child Language*.

[CIT0047] Szagun G. (2001). Learning different regularities: The acquisition of noun plurals by German-speaking children. *First Language*.

[CIT0048] Szagun G. (2004). Learning by ear: On the acquisition of case and gender marking by German-speaking children with normal hearing and cochlear implants. *Journal of Child Language*.

[CIT0049] Tanenhaus M. K., Trueswell J. C., Miller J. L., Eimas P. D. (1995). Sentence comprehension. *Speech, language, and communication*.

[CIT0050] Tomasello M. (2000). Do young children have adult syntactic competence?. *Cognition*.

[CIT0051] Trueswell J. C., Sekerina I., Hill N. M., Logrip M. L. (1999). The kindergarten-path effect: Studying on-line sentence processing in young children. *Cognition*.

[CIT0052] Trueswell J. C., Tanenhaus M. K., Clifton C., Frazier L., Rayner K. (1994). Toward a lexicalist framework of constraint-based syntactic ambiguity resolution. *Perspectives on sentence processing*.

[CIT0053] Warren T., Gibson E. (2002). The influence of referential processing on sentence complexity. *Cognition*.

[CIT0054] Wittek A., Tomasello M. (2005). German-speaking children’s productivity with syntactic constructions and case morphology: Local cues act locally. *First Language*.

